# Reduced levels of circulating endothelial progenitor cells in acute myocardial infarction patients with diabetes or pre-diabetes: accompanying the glycemic continuum

**DOI:** 10.1186/1475-2840-13-101

**Published:** 2014-06-16

**Authors:** Natália António, Rosa Fernandes, Ana Soares, Francisco Soares, Ana Lopes, Tiago Carvalheiro, Artur Paiva, Guilherme Mariano Pêgo, Luís A Providência, Lino Gonçalves, Carlos Fontes Ribeiro

**Affiliations:** 1 Laboratory of Pharmacology and Experimental Therapeutics, Institute for Biomedical Imaging and Life Sciences, Faculty of Medicine, University of Coimbra, Coimbra, Portugal; 2 Cardiology Department, University Hospital Center of Coimbra, Coimbra, Portugal; 3 Center of Ophthalmology and Vision Sciences; Institute for Biomedical Imaging and Life Science, Faculty of Medicine, University of Coimbra, Coimbra, Portugal; 4 Blood and Transplantation Center of Coimbra | Portuguese Institute of Blood and Transplantation, Coimbra, Portugal

**Keywords:** Endothelial progenitor cells, Diabetes, Pre-diabetes, Insulin, Oral antidiabetic drugs, Acute myocardial infarction, Homing

## Abstract

**Background:**

Diabetic patients have a significantly worse prognosis after an acute myocardial infarction (AMI) than their counterparts. Previous studies have shown that the number of circulating endothelial progenitor cells (EPCs) significantly increase early after an AMI in normoglycemic patients. However, it is well known that type 2 diabetes mellitus (DM) is associated with impaired function and reduced circulating EPCs levels. Nonetheless, few studies have analyzed EPCs response of diabetics to an AMI and the EPC response of pre-diabetic patients has not been reported yet. Therefore, we hypothesized that in the acute phase of an AMI, diabetic and pre-diabetics have lower circulating EPCs levels than patients with normal glucose metabolism. We also evaluated the possible capacity of chronic antidiabetic treatment in the recovery of EPCs response to an AMI in diabetics.

**Methods:**

One-hundred AMI patients were prospectively enrolled in the study. Using the high-performance flow cytometer FACSCanto II, circulating EPCs (CD45dimCD34+KDR+ and CD45dimCD133+KDR+ cells) were quantified, within the first 24 hours of admission. In addition, as an indirect functional parameter, we also analyzed the fraction of EPCs coexpressing the homing marker CXCR4.

**Results:**

We found that in the acute phase of an AMI, diabetic patients presented significantly lower levels of circulating CD45dimCD34+KDR+ and CD45dimCD133+KDR+ EPCs by comparison with nondiabetics, with a parallel decrease in the subpopulations CXCR4+ (p < 0.001). Indeed, this study suggests that the impaired response of EPCs to an AMI is an early event in the natural history of DM, being present even in pre-diabetes. Our results, also demonstrated that numbers of all EPCs populations were inversely correlated with HbA1c (r = -0.432, p < 0.001 for CD45dimCD34+KDR+ cells). Finally, this study suggests that previous chronic insulin therapy (but not oral antidiabetic drugs) attenuate the deficient response of diabetic EPCs to an AMI.

**Conclusion:**

This study indicates that there is a progressive decrease in EPCs levels, from pre-diabetes to DM, in AMI patients. Moreover, glycemic control seems to be determinant for circulating EPCs levels presented in the acute phase of an AMI and chronic insulin therapy may probably attenuate the deficit in EPCs pool seen in diabetics.

## Background

It is well recognized that patients with type 2 diabetes mellitus (DM) have accelerated atherosclerosis, increased risk of developing coronary artery disease (CAD) and worse prognosis after an acute myocardial infarction (AMI) [1].

Endothelial progenitor cells (EPCs), a subpopulation of adult stem cells, have emerged as critical to endothelial repair and vascular homeostasis. Although the mechanisms whereby EPCs protect the cardiovascular system are still not fully understood, it has been extensively demonstrated that these bone marrow-derived cells contribute to endothelial repair and postnatal neovascularization [2,3]. EPCs can differentiate into mature endothelial cells and be incorporated into new vessels or act by a paracrine manner, through the secretion of pro-angiogenic growth factors that enhance vascularization mediated by resident endothelial cells and/or promote angiogenesis [2-5].

The number of EPCs in peripheral circulation is generally low, and in normal physiological conditions, these endothelial precursor cells are very rare in blood, but they are mobilized from the bone marrow to the peripheral circulation in response to tissue injury, such as myocardial ischemia [6]. In fact, tissue ischemia is considered the strongest stimulus for EPCs mobilization and it has been shown that their numbers significantly increase in patients with an AMI [7,8]. However, it is well established that diabetic patients present impaired function and reduced numbers of circulating EPCs, reflecting a poor endogenous regenerative capacity that may contribute to the development of vascular complications and to the dismal prognosis associated with this prevalent disease [9-12]. Therefore, it is likely that, in the clinical context of myocardial infarction, diabetic patients also have lower levels of circulating EPCs, but regrettably the data addressing the dynamics of EPCs mobilization in diabetic patients with AMI are scarce. Furthermore, little is known about potential EPCs impairment in pre-diabetic states and no studies are available on the kinetics of EPCs mobilization in pre-diabetic patients with AMI. This is of great importance, since multiple studies have demonstrated that individuals with pre-diabetes are also at increased risk for cardiovascular events [13]. On the other hand, some drugs commonly prescribed in diabetic patients, like statins, angiotensin II receptor blockers (ARBs) and angiotensin-converting-enzyme (ACE)-inhibitors, have been shown to increase the number of EPCs in peripheral blood of patients with stable CAD [14]. However, we have no data available regarding the impact of previous antidiabetic treatment on EPC response to an AMI, in diabetic patients.

In this study, we tested the hypothesis that diabetes and pre-diabetes states were associated with reduced circulating EPCs levels in the acute phase of a myocardial infarction (MI) by comparison with patients with normal glucose metabolism. We also examined the impact of previous antidiabetic treatment on the dynamics of EPCs mobilization in diabetic patients following an AMI.

## Methods

### Study population and selection

**Figure 1:**
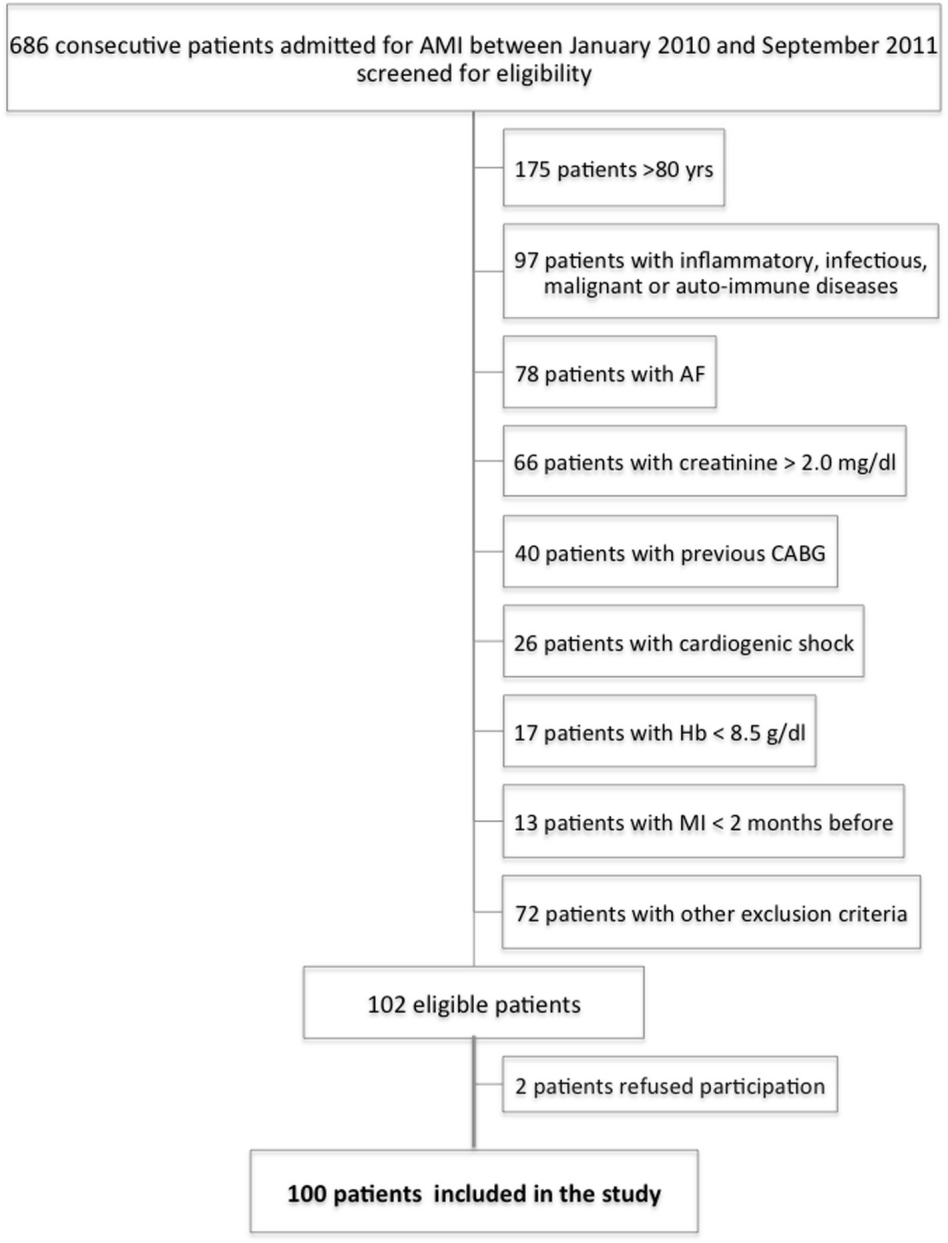
**Flow diagram of patient recruitment.** AF, atrial fibrillation; AMI, acute myocardial infarction; CABG, coronary artery bypass graft; Hb, hemoglobin; MI, myocardial infarction; yrs, years old.

A prospective cohort of 686 consecutive patients hospitalized in a single Coronary Care Unit (CCU) due to myocardial infarction, from 5 January 2009 to 23 September 2011, were screened on admission for inclusion. Screening included an interview, clinical examination, ECG and laboratory assessment. Patients were excluded if they were >80 years old, showed clinical or biochemical evidence of concomitant inflammatory disease, known auto-immune or malignant diseases, severe peripheral arterial occlusive disease, deep vein thrombosis or pulmonary embolism, atrial fibrillation, recent trauma or surgery (<1 month), recent major bleeding requiring blood transfusion (<6 months), renal insufficiency (creatinine > 2.0 mg/dl), anemia (hemoglobin < 8.5 g/dl) or thrombocytopenia (<100 000/L), previous coronary bypass surgery, myocardial infarction within the preceding 2 months, cardiogenic shock, severe valvular disease or congenital heart disease, co-morbidities associated with a life expectancy less than 2 years. A regular use of nonsteroidal anti-inflammatory drugs or anticoagulants, patients with pacemakers, implantable cardioverter defibrillators or resynchronization devices, and excessive alcohol consumption or illicit drugs abuse that may influence EPC kinetics were also exclusion criteria. A total of 100 patients were prospectively included (65% with ST segment elevation myocardial infarction – STEMI and 35% with non-ST segment elevation myocardial infarction - NSTEMI) (Figure 1).

All patients received the standard therapy for the acute phase of MI that included acetylsalicylic acid (ASA), clopidogrel and low-molecular-weight heparin, according to usual hospital practice.

Baseline demographic data, cardiovascular risk factors and previous medications were recorded in all patients. Smoking status was recorded as ever-smoker (past or current) or non-smoker.

Blood samples were collected to assess chemistry (including fasting plasma glucose (FPG) and glycosylated hemoglobin (HbA1C)), total cholesterol, low-density lipoprotein-cholesterol (LDL-C), high-density lipoprotein-cholesterol (HDL-C), triglycerides, high sensitivity C-reactive protein (hs-CRP), creatinine, and hematological parameters in all patients according to standard hospital practice.

The study was approved by the local ethics committee (Approval Number: HUC-23-08). All patients gave written informed consent and research was conducted according to the principles expressed in the Declaration of Helsinki.

### Classification of glucose metabolism status

DM and glucose metabolism disorders were defined according to the American Diabetes Association (ADA) criteria [15,16]. All patients without previously known diabetes underwent an Oral Glucose Tolerance Test (OGTT) on day 4 or 5 of hospitalization. Therefore, patients were classified as having diabetes if they have a FPG ≥126 mg/dl, a 2-h glucose ≥200 mg/dl on OGTT, a A1c ≥ 6.5% or a random plasma glucose ≥200 mg/dl in a patient with classic symptoms of hyperglycemia or hyperglycemic crisis. For patients without diabetes, prediabetes was defined as FPG levels of 100–125 mg/dl (impaired fasting glucose – IFG), 2-hour OGTT glucose level of 140–199 mg/dl (impaired glucose tolerance - IGT) or HbA1c values of 5.7%-6.4%. Patients were classified as having a normal glucose metabolism (NGM) if they have FPG < 100 mg/dl, 2-hour OGTT glucose level < 140 mg/dl and HbA1c <5.7%.

### Quantification of circulating EPCs by flow cytometry

For the identification and quantification of EPCs, we have used a standardized protocol - the modified International Society for Hematotherapy and Graft Engineering (ISHAGE) sequential gating strategy - proposed by Schmidt-Lucke et al. [17]. Briefly, within the first 24 h of CCU admission, 1 ml of whole blood was collected from a forearm vein into EDTA tubes, transported into the cytometry laboratory and processed within 1 to 2 hours of collection. Hence, 150 μl of whole blood were incubated with the following combination of anti-human monoclonal antibodies: 10 μl of anti-CD133 conjugated with allophycocyanin (APC) (Miltenyi Biotec), 5 μl of anti-CD45 conjugated with APC-H7 (Becton Dickinson), 10 μl of anti-KDR (also known as type 2 vascular endothelial growth factor receptor - VEGF-R2) conjugated with phycoerythrin (PE) (Sigma), 10 μl of anti-CD34 conjugated with fluorescein isothiocyanate (FITC) (Becton Dickinson) and 10 μl of anti-CD184 (also known as CXCR4) conjugated with PE-Cyanine 5 (PE-Cy5) (BD Pharmingen) for 30 min at 4°C, in the dark. Red blood cell lysis was performed using FACS Lysing Solution (BDBiosciences) diluted 1:10 (vol/vol) in distilled water and washed with phosphate-buffered-saline (PBS) before flow cytometry acquisition. Data acquisition was performed with a high-performance flow cytometer, a BDBioscience FACSCanto II, which can analyze with high resolution up to eight different fluorescent markers from a large number of events and we used the flow cytometry software Infinicyt 1.5 (Cytognos) for the analysis. According to the used standardized protocol, human circulating EPCs were identified by a minimal antigenic profile that includes at least one marker of stemness/immaturity (CD34 and/or CD133), plus at least one marker of endothelial commitment (KDR). CD45 staining was also performed to exclude leucocytes, as it has been previously demonstrated that only the fraction of CD45dim cells harbours the “true” circulating EPCs [18]. CXCR4, the receptor for stromal cell–derived factor-1 (SDF-1), is a cell surface antigen expressed in EPCs, which plays a key role in their transendothelial migration and homing to sites of vascular injury [19]. Therefore, by analyzing the subpopulation of progenitors coexpressing CXCR4, we could study a functional parameter of EPCs. As isotype controls are known to mask rare cell populations, none were used in this analysis, and baseline fluorescence was determined using unstained cells [20]. Because EPCs are extremely rare events in peripheral blood, additional strategies were applied in order to increase the sensitivity of the method and the accuracy of our work. These included: automatic compensation for minimizing fluorescence spillover, exclusion of dead cells, and use of specific high quality mononuclear antibodies. The total number of acquired events was increased to at least 1 million per sample, which is generally not needed for most other applications of flow cytometry. Circulating EPCs were measured in triplicate from the same patients, revealing a very close correlation (r = 0.87, p < 0.0001). The same trained operator, who was blind to the clinical status of the patients, performed all the cytometric analysis throughout the study.

Four different populations of EPCs were quantified: 1) CD45dimCD133+KDR+ cells; 2) CD45dimCD34+KDR+ cells; 3) CD45dimCD34+CD133+KDR+ triple positive cells; and 4) the subpopulation of CD45dimCD34+KDR+CXCR4+ EPCs.

### Patients follow up for cardiovascular events

All patients were followed up for 24 months after discharge. The following cardiovascular events were recorded: cardiovascular death; nonfatal stroke or transient ischemic attack; re-infarction; unstable angina and re-hospitalization for unstable angina or heart failure. We also analyzed the combined endpoint of cardiovascular death, re-hospitalization for ACS and unplanned PCI – Major Adverse Cardiac Events (MACE). Cardiovascular death was defined as death due to a MI or stroke or documented sudden cardiac death. For patients experiencing more than one acute event, only the first event was considered in the analysis.

### Statistical analysis

Statistical analyses were performed using SPSS software version 20.

Based on previous data, we estimated a 40% reduction in circulating EPCs of diabetics by comparison with nondiabetic patients. Therefore, a minimum sample size of 18 patients in each group would provide 90% power to detect difference in circulating EPCs between diabetic and nondiabetic patients, using a two-sided hypothesis test with a significance level (alpha) of 0.05.

Continuous variables were tested for normal distribution by Kolmogorov–Smirnov test and expressed as mean ± standard deviation or median ± interquartile range for parametric and nonparametric data, respectively. Categorical data are expressed as counts and percentages.

For comparison of continuous data unpaired Student t-tests or ANOVA tests were used when variables were normally distributed and nonparametric Mann–Whitney test or Kruskal-Wallis test for variables without a normal distribution. Categorical variables were compared with the chi-square test or with Fisher exact test as appropriate. The relationship between variables was calculated using Pearson’s or Spearman’s correlation coefficient, whichever appropriate. Multivariate linear regression analysis was used to assess the relationship between circulating EPCs levels and HbA1c, after adjustment for confounding variables. Kaplan-Meier survival analyses were performed to evaluate time-dependent outcomes. Differences between pairs of survival curves were tested by the log-rank test. For all analyses, a 2-sided value of P < 0.05 was considered statistically significant.

## Results

### Characteristics of the study population

There were 38 patients with DM, 13% of them with newly diagnosed DM. Overall, diabetics had similar age and cardiovascular risk factors as nondiabetic patients, except for hypertension that was significantly more frequent in diabetics (Table 1). Additionally, they tended to have more frequently previously known CAD and were more often medicated with ASA, ACE-inhibitors and ARB as well as oral hypoglycemic agents and insulin before admission than nondiabetics. As expected, diabetics had significantly higher levels of admission glycemia, fasting glycemia and HbA1c and also presented higher total cholesterol and LDL-cholesterol than nondiabetics.

**Table 1:** Comparison of clinical characteristics between diabetic and nondiabetic patients

	**Non-diabetics (N = 62)**	**Type 2 diabetics (N = 38)**	**p value**
Age (years)*	59.8 ± 10.3	61.5 ± 11.0	0.300
Male gender (%)	90.3	89.5	0.891
BMI (Kg/m^2^)*	27.9 ± 4.4	29.2 ± 6.9	0.251
Previous CAD (%)	14.5	31.6	0.075
Previous MI (%)	11.3	18.4	0.319
Type of MI			
STEMI vs NSTEMI (%)	66.1/33.9	63.2/36.8	0.762
Cardiovascular risk factors			
Hypertension (%)	56.5	84.2	0.004
Smoking habits (%)	61.3	47.4	0.215
Family history (%)	37.1	28.9	0.492
Hyperlipidemia (%)	71.1	82.3	0.189
Physical inactivity (%)	56.5	60.5	0.689
Previous cardiovascular or antidiabetic drugs			
Statins (%)	29.0	31.6	0.825
ASA (%)	19.4	42.1	0.021
ACEI (%)	12.9	36.8	0.007
ARB (%)	12.9	31.6	0.038
Beta-blockers (%)	9.7	21.1	0.141
Insulin (%)	0.0	26.3	<0.001
Oral hypoglycemic (%)	0.0	65.8	<0.001
Baseline laboratory			
Admission Troponin I (μg/L)^§^	0.7 ± 5.8	1.5 ± 3.6	0.798
Peak Troponin I (μg/L)^§^	55.4 ± 71.6	56.7 ± 64.7	0.793
HbA1C (%)^§^	5.6 ± 0.5	7.1 ± 2.2	<0.001
Admission glycemia (mg/dl)^§^	109.0 ± 31.0	206.5 ± 110.8	<0.001
First fasting glycemia (mg/dl)^§^	103.0 ± 24.5	156.0 ± 52.5	<0.001
Total cholesterol (mg/dl)*	178.5 ± 59.0	211.7 ± 54.9	0.007
LDL cholesterol (mg/dl)*	113.2 ± 39.6	145.3 ± 44.3	<0.001
HDL cholesterol (mg/dl)^§^	40.2 ± 9.5	38.7 ± 12.9	0.164
Triglycerides (mg/dl)^§^	138.5 ± 109.5	148.0 ± 88.5	0.801
Uric acid (mg/dl)*	5.6 ± 1.3	6.2 ± 1.4	0.096
Baseline creatinine (mg/dl)^§^	0.8 ± 0.3	0.9 ± 0.4	0.123
Baseline hemoglobin (g/dl)*	14.8 ± 1.4	14.4 ± 1.2	0.200
Admission hs-CRP (mg/dl)*	0.9 ± 1.3	1.0 ± 1.4	0.872
LVEF (%)*	52.6 ± 9.6	50.0 ± 11.8	0.104
Hospital length of stay^§^	5.4 ± 2.6	5.9 ± 3.0	0.424

ACEI, Angiotensin-Converting Enzyme Inhibitors; ARB, Angiotensin II receptor blockers; ASA, acetylsalicylic acid; CAD, coronary artery disease; hs-CRP, high sensitivity C-reactive protein; LVEF, left ventricular ejection fraction; MI, myocardial infarction; STEMI, ST elevation myocardial infarction; NSTEMI, non-ST elevation myocardial infarction.

*mean ± SD.

^§^median ± interquartile range.

There were no significant differences in MI presentation (STEMI versus NSTEMI), left ventricular function or renal function between groups (Table 1).

There were no significant differences in the extent of coronary atherosclerosis, number of stents deployed or other cath lab parameters between diabetics and nondiabetics (Table 2).

**Table 2:** Comparison of catheterization lab data between diabetics and nondiabetics

	**Non-diabetics (N = 62)**	**Type 2 diabetics (N = 38)**	**p value**
Catheterization during hospitalization (%)	94.7	96.8	0.614
Normal coronaries (%)	5.0	0.0	0.289
1-vessel disease (%)	41.7	41.7	1.000
2-vessel disease (%)	26.7	30.6	0.682
3-vessel disease (%)	26.7	27.8	0.906
Left main disease (%)	6.7	5.7	0.854
LAD disease (%)	69.5	80.6	0.235
PCI before EPCs evaluation (%)	72.6	68.4	0.656
Complete revascularization before EPCs evaluation (%)	44.9	45.3	0.912
Number of stents deployed before EPCs evaluation	1.6 ± 1.1	1.8 ± 1.2	0.649

LAD; left anterior descending, PCI; percutaneous coronary intervention.

### Reduction of circulating EPCs in diabetic patients

**Figure 2:**
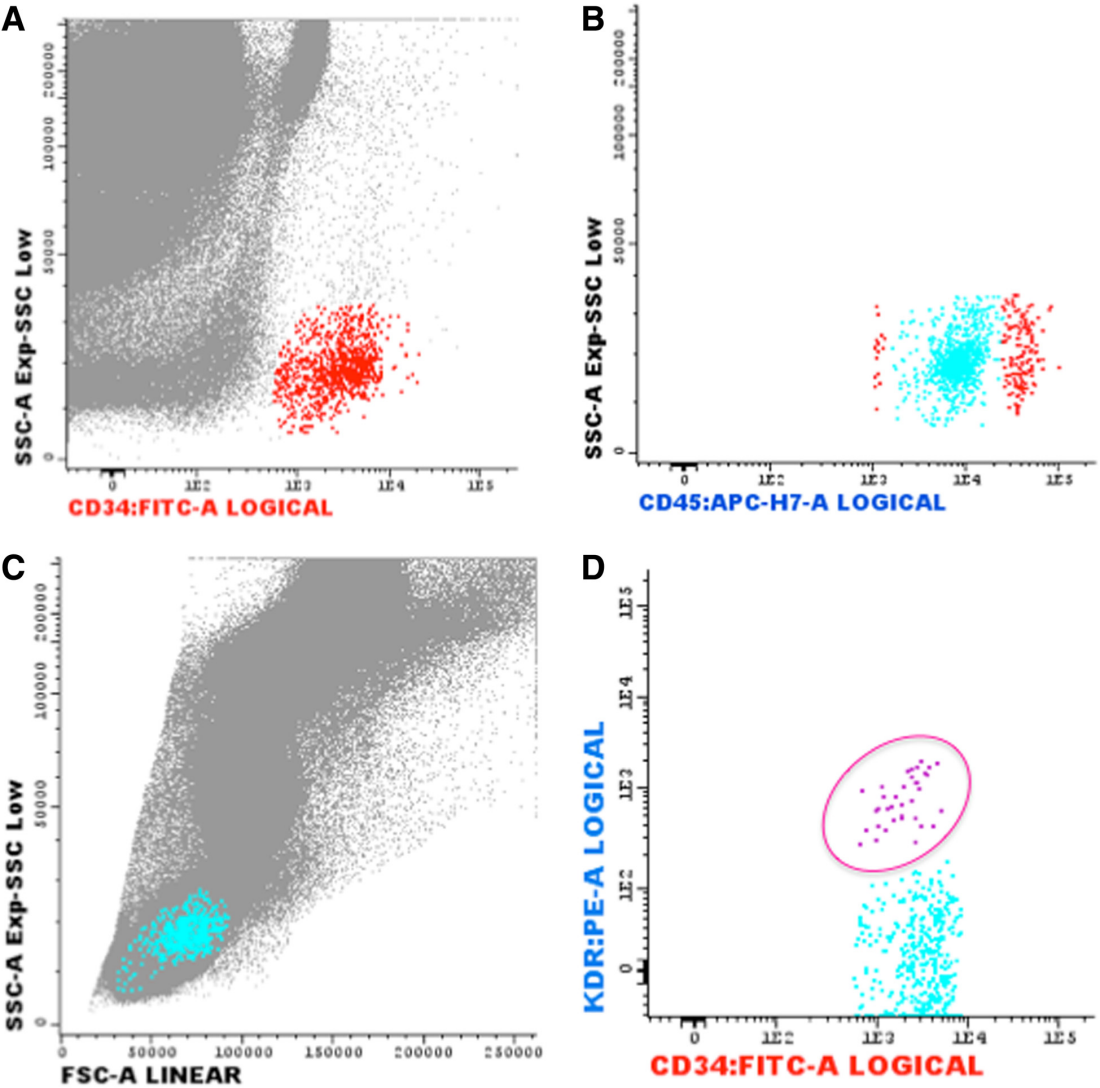
**Representative flow cytometry quantification of CD45low/CD34+/KDR+ EPCs in a a nondiabetic patient with STEMI. (A)** The cluster of CD34+ cells (red points) was analyzed using FITC-labeled antibodies against CD34 vs. SSC. **B)** CD45dim subset analysis was conducted using APC-H7-labeled antibodies against CD45 vs SSC. At this step, events forming a cluster of characteristic low SSC and low CD45 fluorescence (SSClowCD45dim cells) expressed CD34+CD45dim cells (light blue events). **C)** The events fulfilling previous criteria were then displayed on a forward light scatter (FSC) vs. SSC dot plot to confirm that the selected cells fall into the lymphocyte region. **D)** Finally, CD45dimCD34+KDR+ endothelial progenitor cells (pink cells) are deducted in the dot plot of PE-labeled antibodies against KDR vs CD34.

**Figure 3:**
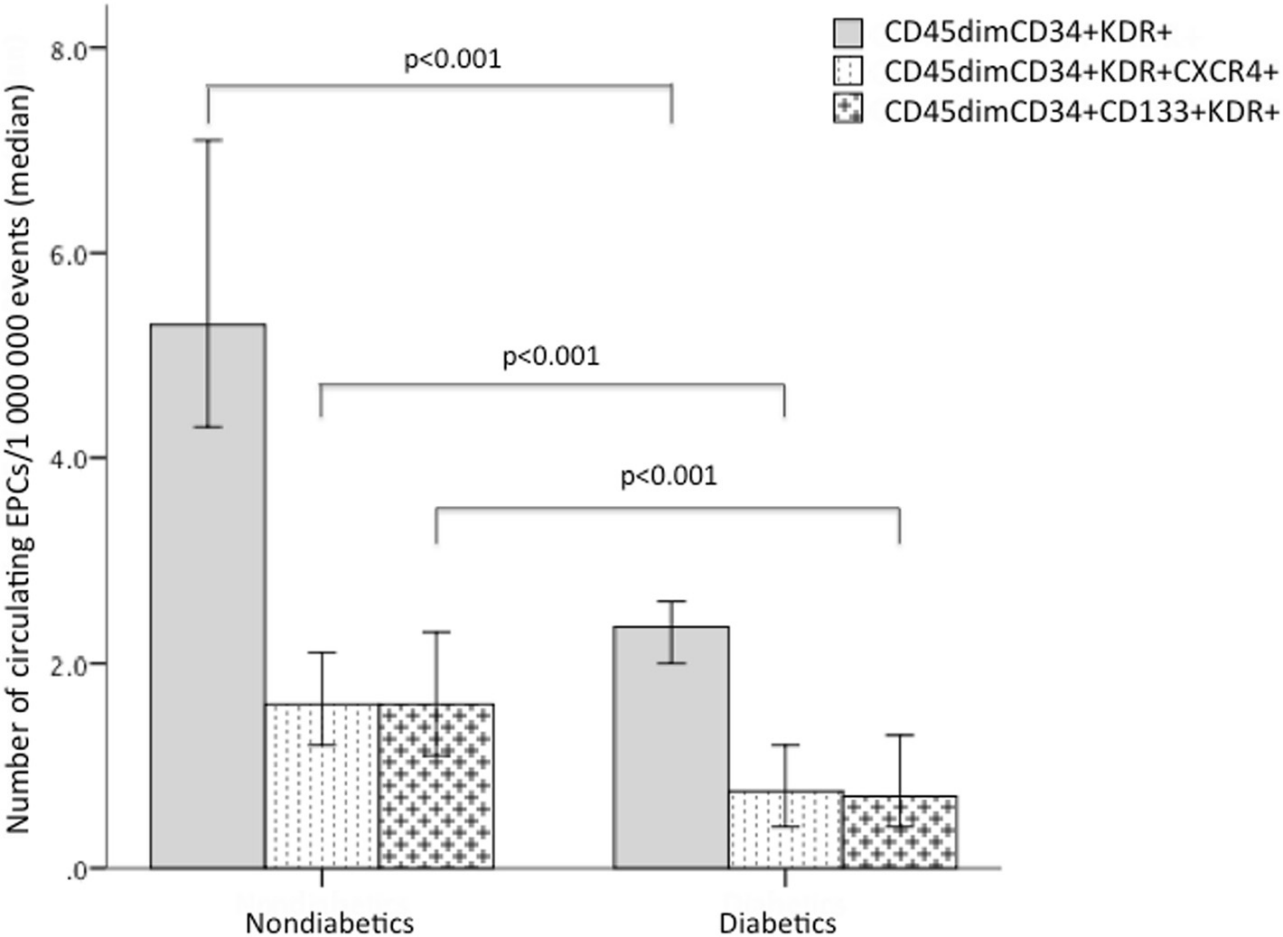
**Comparison of circulating EPCs levels between diabetics and nondiabetics.** Bars represent median and error bars interquartile range of circulating EPCs numbers quantified by flow cytometry. Mann Whitney U test was used for the comparison between diabetic and nondiabetic EPCs levels.

Circulating EPCs levels were expressed for one million cytometric events (Figure 2). Diabetic patients had circulating numbers of CD45^dim^CD34+KDR+ cells reduced by 63% when compared with nondiabetics, with a parallel decrease in the subpopulation CXCR4+ (Table 3, Figure 3). There was also a significant reduction in the more immature population of CD45dimCD34+CD133+KDR+ EPCs to around half the levels of nondiabetics, and numbers of its precursors CD45dimCD133+KDR+ in peripheral circulation were also significantly decreased. The subpopulation coexpressing the homing marker CXCR4 (CD45dimCD133+KDR+CXCR4+) was also significantly reduced in diabetics (Table 3).

**Table 3:** Comparison of circulating EPCs levels between diabetics and nondiabetics

	**Non-diabetics (n = 62)**	**Type 2 diabetics (n = 38)**	**p value**
Time from PCI to blood sampling (hours)	13.8 ± 14.7	11.6 ± 11.7	0.649
CD34+ cells/10^6^ WBC	228.8 ± 136.7	197.0 ± 115.2	0.098
CD133+/10^6^ WBC	54.4 ± 35.7	36.0 ± 18.0	0.020
CD45dimCD34+KDR+ cells/10^6^ WBC	6.2 ± 3.0	2.3 ± 0.9	<0.001
CD45dimCD34+KDR+CXCR4+ cells/10^6^ WBC	1.8 ± 1.1	0.8 ± 0.7	<0.001
CD45dimCD34+CD133+KDR+ cells/10^6^ WBC	2.1 ± 1.1	1.0 ± 0.8	<0.001
CD133+KDR+/10^6^ WBC	4.6 ± 2.9	3.1 ± 1.6	<0.001
CD133+KDR+CXCR4+/106 WBC	3.5 ± 1.9	2.0 ± 1.2	<0.001

PCI, percutaneous coronary intervention; WBC, white blood cells.

### Circulating EPCs levels across the different disorders of glucose metabolism

Upon OGTT, 24 of the nondiabetic patients had pre-diabetes (29.2% with impaired fasting glucose - IFG, 58.3% with impaired glucose tolerance – IGT and 12.5% with both disorders of glucose metabolism).

Circulating CD45dimCD34+KDR+ EPCs decreased as a continuum from NGM to DM, as there was a reduction of approximately 40% in patients with pre-diabetes as compared with NGM patients (p = 0.018) and there was an additional reduction of these EPCs of about 40% (p = 0.042) when diabetics were compared with patients with pre-diabetes (Table 4). Nonetheless, the population of more immature progenitor cells (CD45dimCD133+KDR+) and the subpopulations coexpressing the CXCR4 marker (CD45dimCD34+KDR+CXCR4+ and CD45dimCD133+KDR+CXCR4+) were not significantly reduced in pre-diabetic patients by comparison with NGM patients (5.4 ± 2.4 vs 3.9 ± 2.8, p = 0.314; 1.8 ± 0.9 vs 1.3 ± 1.2, p = 0.175; and 3.5 ± 2.1 vs 3.2 ± 1.3, p = 0.290, respectively), whereas a significant reduction was apparent from pre-diabetic to diabetic patients on these cells levels (p = 0.022; p = 0.045 and p = 0.015, respectively) (Table 4).

**Table 4:** Comparison of circulating EPCs levels between the different glucose metabolism status

	**NGM (n = 38)**	**Pre-diabetes (n = 24)**	**Diabetes (n = 38)**	**p value**
CD34+ cells/10^6^ WBC	417.3 ± 266.9	225.4 ± 97.5	176.5 ± 148.8	0.006
CD133+/10^6^ WBC	41.5 ± 23.7	34.1 ± 21.2	34.4 ± 19.2	0.101
CD45dimCD34+KDR+ cells/10^6^ WBC	7.0 ± 3.5	4.3 ± 2.7	2.4 ± 1.2	<0.001
CD45dimCD34+KDR+CXCR4+ cells/10^6^ WBC	1.8 ± 0.9	1.3 ± 1.2	0.8 ± 0.7	0.002
CD45dimCD34+CD133+KDR+ cells/10^6^ WBC	1.7 ± 1.0	1.3 ± 1.1	0.7 ± 0.6	0.001
CD133+KDR+/10^6^ WBC	5.4 ± 2.4	3.9 ± 2.8	3.0 ± 1.9	0.002
CD133+KDR+CXCR4+/10^6^ WBC	3.5 ± 2.1	3.2 ± 1.3	2.0 ± 1.4	0.002

NGM, normal glucose metabolism; WBC, white blood cells.

### Circulating EPCs numbers according to previous antidiabetic treatment

**Figure 4:**
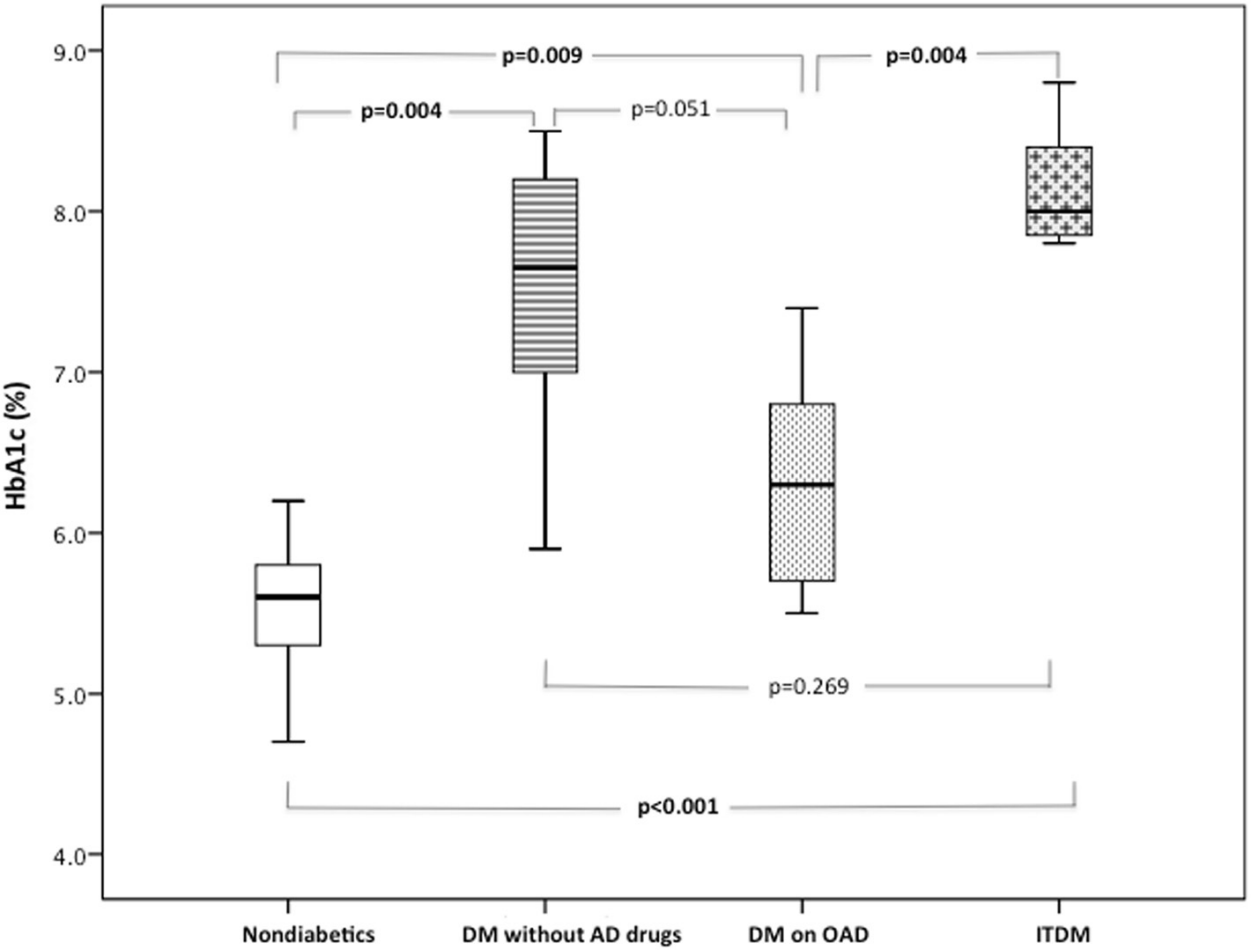
**Glycosylated hemoglobin levels according to chronic antidiabetic treatment.** Box plots represent the interquartile range of values, the horizontal lines show the median, and whiskers represent the maximum and minimum values. In order to find between which antiadiabetic treatment categories there were significant differences, we compared two by two groups using the Mann Whitney U test.

**Figure 5:**
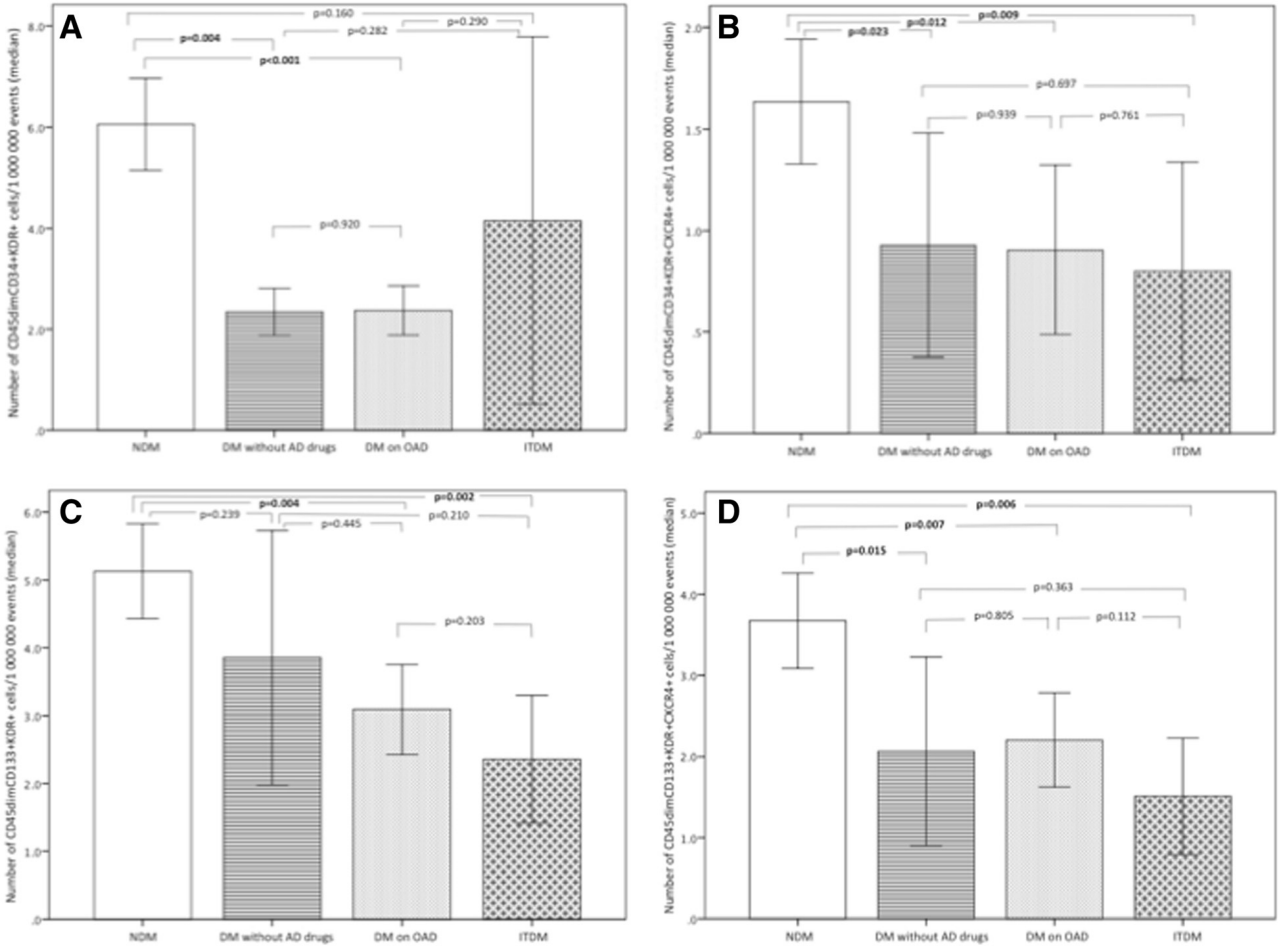
**Comparison of circulating EPCs levels between nondiabetic patients and diabetics under different antidiabetic treatments. (A)** Circulating numbers of CD45dimCD34+KDR+ cells; **(B)** Circulating numbers of CD45dimCD34+KDR+CXCR4+ cells; **(C)** Circulating numbers of CD45dimCD133+KDR+ cells and **(D)** Circulating numbers of CD45dimCD133+KDR+CXCR4+ cells. Bars represent median and error bars interquartile range of circulating EPCs numbers quantified by flow cytometry. We compared two by two groups using the Mann Whitney U test, to evaluate between which antiadiabetic treatment category there were significant differences.

Regarding the antidiabetic strategy before admission, there were 53% of diabetic patients on oral hypoglycemic drugs, 26% insulin-treated diabetics, and 21% of patients who were not taking any antidiabetic drug (because they were on diet-only therapy or new onset DM was diagnosed during hospitalization). As expected, diabetes duration was significantly longer in insulin-treated patients (13.5 ± 9.8 years versus 6.8 ± 5.0 in patients on oral hypoglycemic drugs versus 1.7 ± 1.2 in diabetics not receiving any antidiabetic drug, p = 0.001). Insulin-treated DM (ITDM) patients and diabetics not previously treated with antidiabetic drugs presented a worse glycemic control as compared with patients on oral hypoglycemic drugs (Figure 4).Numbers of CD45dimCD34+KDR+ EPCs were significantly reduced in diabetic patients previously treated with oral antidiabetic drugs and in diabetics not taking any hypoglycemic drug when compared with nondiabetic patients (Figure 5, A). However, despite the worse glycemic control of diabetics on chronic insulin, their CD45dimCD34+KDR+ EPCs levels were not significantly reduced compared to that of nondiabetic patients (p = 0.160) (Figure 5-A). Regarding the subpopulation of CD45dimCD34+KDR+ cells also expressing the homing marker CXCR4+, all diabetes treatment categories presented significantly decreased circulating levels by comparison with nondiabetic patients (Figure 5-B). Circulating CD45dimCD133+KDR+ cell levels showed a progressive decline from nondiabetics, untreated DM, DM on oral hypoglycemic drugs and finally, ITDM, with patients receiving insulin and patients on oral hypoglycemic drugs presenting significantly lower levels as compared with nondiabetics (p = 0.002 and p = 0.004, respectively) (Figure 5-C). Circulating levels of the CD45dimCD133+KDR+CXCR4+ subpopulation were also significantly lower in all diabetic treatment categories than in nondiabetic patients (Figure 5-D).

### Impact of glycemic control on EPCs levels

**Figure 6:**
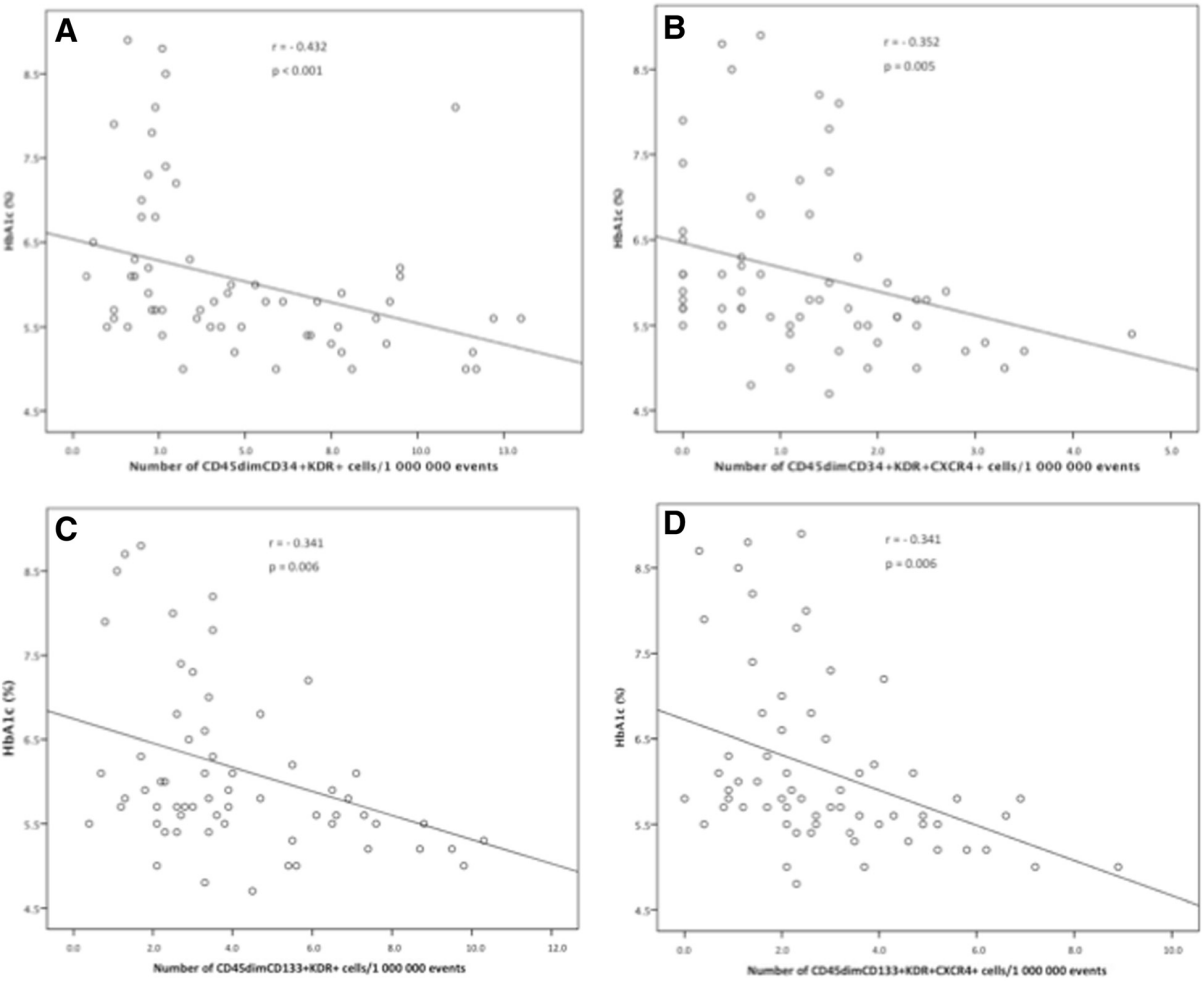
**Relationship between levels of circulating EPCs and HbA1c.** Scatter plots showing significant negative relationship relationship (Pearson correlation) between HbA1c and circulating levels of: **A)** CD45dimCD34+KDR+ cells; **B)** CD45dimCD34+KDR+CXCR4+ cells; **C)** CD133+KDR+ cells; **D)** CD133+KDR+CXCR4+ cells.

There were significant negative correlations between levels of circulating CD45dimCD34+KDR+ (Figure 6, A), CD45dimCD133+KDR+ progenitors (Figure 6, C), their CXCR4+ subpopulations (Figure 6, B and D) and HbA1c. CD45dimCD34+KDR+EPCs and their subpopulation CD45dimCD34+KDR+CXCR4+ were also inversely correlated with fasting glycemia (r = - 0.371, p < 0.001 and r = - 0.213, p = 0.046, respectively). Nonetheless, EPCs levels were not correlated with DM duration. Levels of circulating CD45dimCD34+KDR+ and CD45dimCD133+KDR+ progenitors were also negatively correlated with age (r = - 0.285, p = 0.007 and r = - 0.343, p = 0.001, respectively).

Remarkably, correlations with HbA1c remain significant even after adjustment for age, gender, hypertension, LDL-cholesterol, family history of CAD, smoking habits and physical inactivity (Table 5).

**Table 5:** Multivariate regression analysis assessing the correlation between HbA1c and circulating progenitor cells levels, after adjustment for other cardiovascular risk factors than diabetes

**Variable**	**CD45dimCD34+KDR+ levels**		**CD45dimCD34+KDR+CXCR4+ levels**		**CD133+KDR+ levels**		**CD133+KDR+CXCR4+ levels**	
	**Standard coefficient (ß)**	**p**	**Standard coefficient (ß)**	**p**	**Standard coefficient (ß)**	**p**	**Standard coefficient (ß)**	**p**
HbA1c	-0.308	0.019	-0.260	0.031	-0.342	0.009	-0.416	0.001
Age	-0.188	0.217	-0.107	0.482	-0.254	0.090	-0.052	0.720
Gender	0.208	0.067	0.207	0.119	0.044	0.740	0.158	0.233
Hypertension	-0.071	0.597	-0.075	0.579	0.025	0.855	-0.015	0.905
LDL-cholesterol	-0.057	0.683	-0.047	0.727	-0.176	0.202	-0.148	0.267
Family history of CAD	-0.129	0.361	-0.170	0.202	0.081	0.560	0.078	0.563
Smoking habits	0.003	0.985	-0.204	0.188	-0.086	0.563	-0.133	0.369
Physical inactivity	-0.203	0.139	-0.080	0.556	-0.057	0.657	-0.167	0.180
Adjusted R^2^	0.264	…	0.256	…	0.246	…	0.247	…
Significance (ANOVA)	…	0.032	…	0.040	…	0.046	…	0.033

CAD, coronary artery disease; HbA1c, hemoglobin A1c; LDL, low density lipoprotein.

### Prognostic impact of EPCs

Clinical outcomes during the 24 months follow-up period are represented in Table 6.

**Table 6:** Comparison of clinical outcomes after AMI between diabetics and nondiabetics

	**Nondiabetics (N = 62)**	**Diabetics (N = 38)**	**Odds ratio**	**P value**
Cardiovascular mortality (%)	1.6	7.9	5.2	0.120
Stroke or TIA (%)	0	5.4	-	0.064
Re-infarction (%)	3.3	0	-	0.266
Unstable Angina (%)	3.3	18.9	6.9	0.009
Re-hospitalization for UA or HF (%)	8.1	29.7	4.8	0.005
MACE (%)	9.8	31.6	4.2	0.006

AMI, acute myocardial infarction; HF, heart failure; MACE, Major Adverse Cardiac Events; TIA, transient ischemic accident; UA, unstable angina.

**Figure 7:**
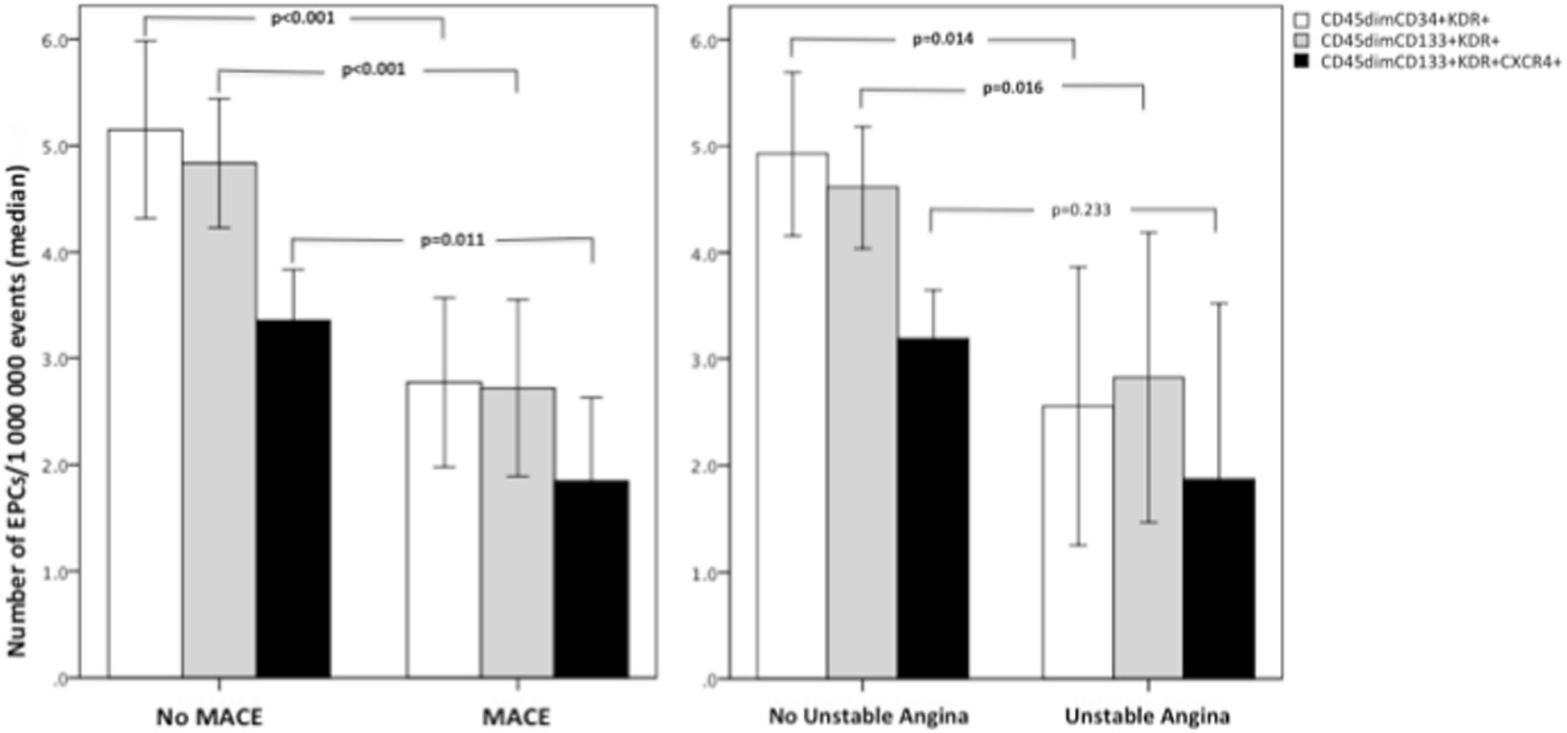
**Comparison of baseline EPCs levels between patients with or without cardiovascular events during the 2-year follow-up period.** Bars represent median and error bars interquartile range of circulating EPCs numbers, quantified by flow cytometry within the first 24 h of admission. We compared EPCs levels between patients with versus without MACE (left panel) and between patients with versus without unstable angina (right panel), using the Mann Whitney U test.

**Figure 8:**
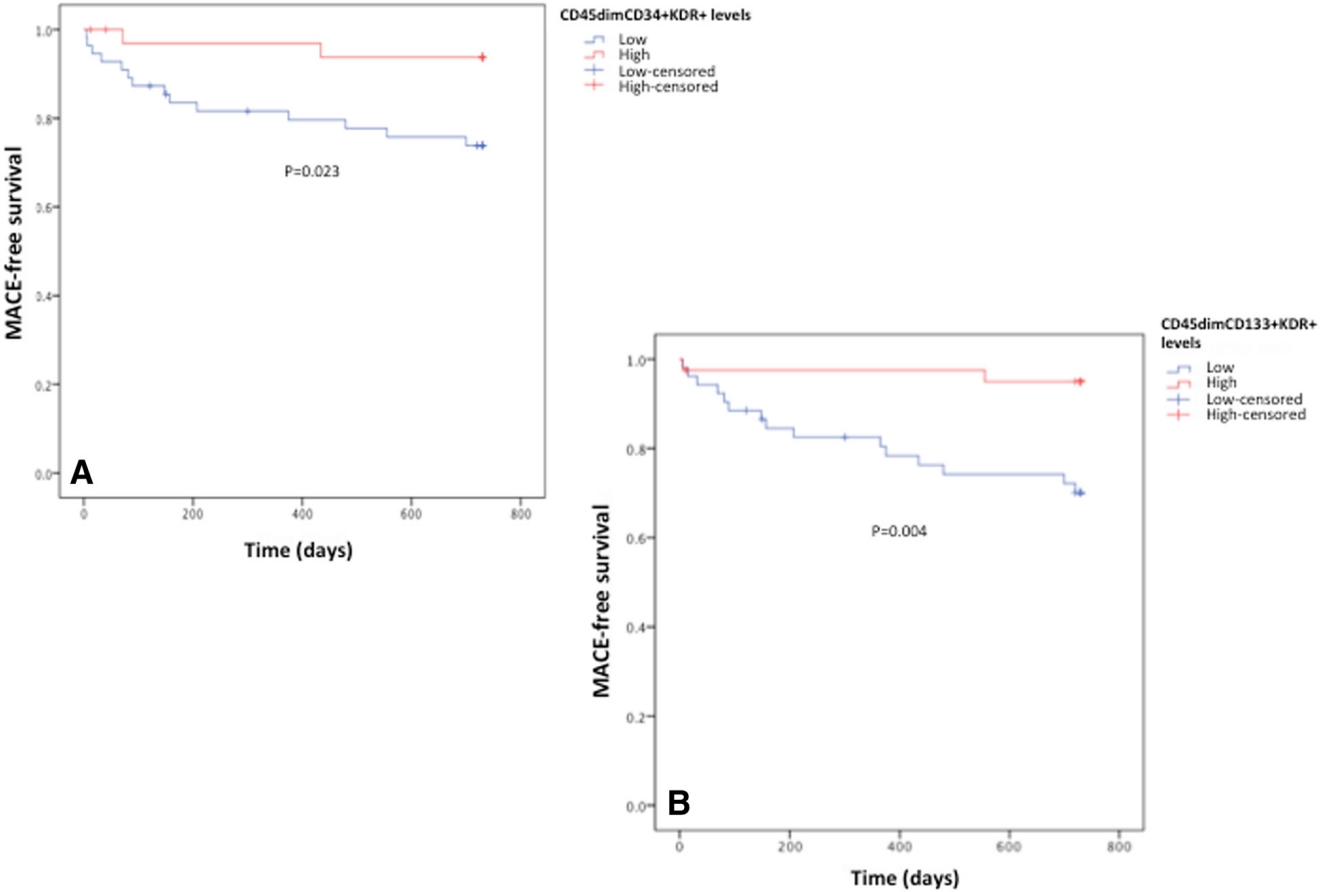
**Kaplan-Meier MACE-free survival curves following an AMI, according to circulating EPCs levels presented in the acute phase. A)** Comparison of MACE-free survival curves between patients with levels of CD45dimCD34+KDR+ cells above (red line) or below (blue line) the median. **B)** Comparison of MACE-free survival curves between patients with levels of CD133+KDR+ cells above (red line) or below (blue line) the median level. The p value was determined by log-rank test.

There were no significant differences in re-infarction, nonfatal stroke/transient ischemic attack or cardiovascular mortality rates between groups. However, the occurrence of unstable angina, the composite endpoints MACE and re-hospitalization for unstable angina or heart failure were significantly higher in diabetics, with the following odds ratios 6.89 (95% CI, 1.35-35.19), 4.23 (95% CI 1.43-12.53) and 4.82 (95% CI 1.52-15.30), respectively.Regarding baseline circulating EPCs levels, patients with unstable angina, unplanned PCI or MACE during follow-up presented significantly lower levels of CD45dimCD34+KDR+ and CD45dimCD133+KDR+ EPCs. Levels of the CD45dimCD133+KDR+CXCR4+ EPCs subpopulation were also significantly reduced, at baseline, in patients who underwent unstable angina or MACE during the 2-year follow-up period (Figure 7). Additionally, the Kaplan–Meier survival curves for freedom from MACE according to EPCs levels showed a significantly lower event-free survival rate in patients with lower EPCs levels in the early phases of AMI (log-rank test, p = 0.023 for CD45dimCD34+KDR+ EPCs and log-rank test, p = 0.004 for CD45dimCD133+KDR+ cells) (Figure 8).

## Discussion

There were four major findings in the present study. First, we confirmed that, in the acute phase of a MI, diabetic patients present dramatically reduced levels of circulating EPCs by comparison with nondiabetics. Second, this study showed for the first time that even pre-diabetes reduces EPCs response to an AMI, since EPCs levels were significantly reduced in pre-diabetics and further reduced in diabetics as compared with patients with NGM. Third, previous chronic insulin therapy (but not oral antidiabetic drugs) seems to attenuate the deficit in circulating EPCs seen in diabetic patients with an AMI. Finally, we have demonstrated that the degree of glycemic control is an important determinant of circulating EPCs numbers in the setting of an AMI.

An AMI is a recognized pathological stimulus for EPCs mobilization. In fact, patients with AMI present significantly increased numbers of circulating EPCs as compared with control subjects or with patients with stable angina [8]. It has been shown that circulating EPCs increase immediately after the onset of an AMI, with a subsequent peak at day 5 and a rapid decline thereafter, normalizing within 2 months [21,22]. Circulating EPCs constitute a key endogenous repair mechanism to counteract ongoing endothelial cell injury, replace dysfunctional endothelium, and enhance tissue repair after ischemic vascular injury [23]. Of note, depletion of circulating EPCs pool and impaired migratory activity of these progenitor cells have been shown to be predictive of future adverse cardiovascular events [24,25]. In accordance with these previous studies, our work showed that freedom from MACE following an AMI was significantly poorer in patients with lower baseline EPCs levels.

It has been extensively demonstrated that patients with DM have a profound reduction of EPCs levels in peripheral blood, which has been correlated with the high cardiovascular morbidity and mortality associated with diabetes [10,26]. Additionally, reduced EPCs numbers have been independently associated with impaired myocardial function in diabetic patients [27]. Fadini et al. have demonstrated in diabetic animals, a deficient EPCs mobilization and impaired compensatory angiogenesis after hindlimb ischaemia-reperfusion injury [28]. However, in the clinical setting of AMI, and despite the important vascular protective role of EPCs, to date, only three clinical studies have studied the dynamics of EPCs mobilization in diabetic patients [21,22,29]. In those studies, circulating EPCs levels were decreased in diabetics [21,22] (or hyperglycemic patients, in the Marfella et al. study) [29] compared with non-diabetic patients immediately after the onset of AMI (day 1). Moreover, it has been demonstrated that the peak level of circulating EPCs was delayed in diabetic patients compared with that of nondiabetic patients (from day 5 in nondiabetic patients to day 7 in diabetic patients) [21,22]. Consistent with these previous studies, the present work confirmed that circulating EPCs levels were strikingly reduced in the early phases of an AMI in diabetic patients as compared with nondiabetic patients. Of note, this importantly reduction in EPCs levels seen in diabetic patients does not seem justified by differences in myocardial ischemia or different coronary revascularization procedures between groups, as values of troponin I (a highly specific marker of myocardial injury) and coronary revascularization were similar in diabetic and nondiabetic patients.

It has become evident that circulating EPCs numbers were inversely correlated to the severity of CAD [30,31]. However, in the present study the huge difference in EPCs levels between AMI diabetics and nondiabetics cannot be explained by differences in CAD severity, since there were no significant differences in the extension of coronary stenosis between both groups.

A large body of evidence links classical cardiovascular risk factors, such as hypertension, with reduction in circulating EPCs [32]. In this study population, diabetics presented a significantly higher prevalence of hypertension that could exacerbate the difference in EPCs levels as compared with nondiabetics. However, diabetics were also more frequently treated with drugs that recognizably increase circulating EPCs numbers, such as ACE-inhibitors and ARBs, what would counterbalance the possible reduction on EPCs numbers due to the higher prevalence of hypertension [14].

EPCs can be identified on the basis of the expression of surface markers, by flow cytometry, a method considered the gold standard for the quantification of these cells in peripheral blood [33]. Of note, there are no unique or specific surface antigen that can be used to identify circulating EPCs. Therefore, FACS protocols must use the combination of various membrane markers for EPCs quantification. In the present work, we used a standardized polychromatic FACS protocol based upon the detection of CD34 (an adhesion molecule expressed mainly on haematopoietic stem cells) [34], CD133/AC133 (a surface marker expressed in an immature subset of EPCs, which share more characteristics of stem/progenitor cells) [35], KDR/VEGF-R2 (a typical endothelial marker) [36], CXCR4/CD184 (a homing marker) [19] and CD45dim (critical to exclude myeloid cells and because it has been previously demonstrated that only the fraction of CD45dim cells harbors the “true” circulating EPCs) [17,18]. Importantly, there are no studies in the literature that have attempted to quantify, at the same time, both CD45dimCD34+KDR+EPCs and the more immature population of CD45dimCD133+KDR+ progenitors in patients with AMI. Thus, until now there has been no data available on the relation between these 2 populations in diabetics with an AMI, which would be important to elucidate the mechanisms underlying their impaired response. In this study, we showed for the first time that, not only CD45dimCD34+KDR+ but also 2the more immature precursors CD45dimCD34+CD133+KDR+ and CD45dimCD133+KDR+ were significantly reduced in diabetic AMI patients by comparison with nondiabetics. Based on these results, it is tempting to speculate that EPCs reduction in diabetes was due, at least in part, to impaired bone marrow mobilization. Because, if the reduction in EPCs levels was motivated by a decrease in survival alone it would be expected to have reduced levels of CD45dimCD34+KDR+ but increased, or at least normal, levels of the more immature population of CD45dimCD133+KDR+ cells, due to positive feedback stimulation of bone marrow recruitment. What we verified here was that the reduction in the more mature EPCs population was not accompanied by the expected up regulation of the more immature ones. In fact, despite the reduction in CD45dimCD34+KDR+ levels, CD45dimCD133+KDR+ and CD45dimCD34+CD133+KDR+ precursors were also reduced, pointing to impairment in recruitment mechanisms.

Besides the reduction in EPCs counts, we found that the fraction of EPCs coexpressing the homing receptor CXCR4 were also significantly reduced in diabetic AMI patients what may represent an impaired homing capacity of these cells to sites of vascular damage. In fact, CXCR4, the only known receptor for SDF-1, has been reported to play an important role in EPCs homing [19]. Moreover, CXCR4/SDF-1 interaction influences proliferation and mobilization of EPCs from the bone marrow [37]. Since functional study of EPCs, in large populations, with in vitro assays is prohibitively expensive and time consuming, the analysis by flow cytometry of EPCs coexpressing CXCR4 may provide a promising alternative parameter to assess EPCs function. This is the first study to show a reduction in numbers of EPCs coexpressing CXCR4 in diabetic patients with AMI compared with AMI nondiabetics. It is probable that this down regulation in CXCR4+ cells denotes a homing impairment, which in addition to the markedly reduction in circulating EPCs levels may contribute to the worsened outcome post-AMI observed in diabetics.

Pre-diabetes is a general term that refers to an intermediate stage between NGM and overt DM, including IFG and IGT. These disorders of glucose metabolism confer an increased risk for developing both DM and cardiovascular events [13,15,38]. In the present study we have found that CD45dimCD34+KDR+ EPCs were significantly lower in pre-diabetic patients and further reduced in those with DM, as compared with individuals with NGM, suggesting that the reduction in the more mature EPCs population follows the continuum of DM development. These findings suggest that circulating EPCs reduction is an early event in the natural history of DM, what is in accordance to a previous work of Fadini et al. [39]. That study has shown, in individuals from a metabolic outpatient clinic, that circulating CD34+KDR+ cells present a progressive decline from NGM, to prediabetics and diabetic patients and that both fasting and post-challenge glucose were inversely related to circulating CD34+KDR+ EPCs levels [39]. Our work further extends these findings by the quantification of more immature EPCs populations and the study of homing function by the analysis of CXCR4+ subpopulations. Interestingly, we verified that CD45dimCD133+KDR+ EPCs and both subpopulations of CXCR4+ EPCs (CD45dimCD34+KDR+CXCR4+ and CD45dimCD133+KDR+CXCR4+ cells) were not significantly reduced in pre-diabetic AMI patients, compared to patients with NGM. One possible explanation for this divergent influence on different EPCs populations is that pre-diabetes reduces EPCs survival (with subsequent reduction CD45dimCD34+KDR+ EPCs levels) but, does not impair neither bone marrow recruitment of EPCs (leading to no differences in levels of CD45dimCD133+KDR+ EPCs) nor homing processes (explaining the normal proportion of EPCs coexpressing CXCR4).

Previous *in vitro* and several animal studies have demonstrated that insulin therapy has a protective role over EPCs function [40-42]. More recently, Marfella et al. have demonstrated, in hyperglycemic patients with AMI, that EPCs levels increased after insulin infusion for intensive glycemic control [29]. Regarding oral antidiabetic drugs, several clinical studies have shown that PPAR-γ agonists, such as rosiglitazone and pioglitazone and also DPP-4 inhibitor sitagliptin increase EPCs levels and improve their function in diabetic patients [43-45]. However, little is known about the molecular mechanisms that regulate the beneficial effects of all these antidiabetic drugs over EPCs.

Importantly, evidence demonstrates that the degree of hyperglycemic control in diabetic patients is closely related to circulating EPCs levels [46,47]. However, despite the obvious interest to know the impact of chronic antidiabetic therapy on EPCs response of diabetic patients to an AMI, until now there have been no studies in the literature addressing this subject. Therefore, in the present work we have studied this issue and verified that, despite the longer DM duration and the worse glycemic control, insulin treated patients presented levels of CD45dimCD34+KDR+ EPCs that tended to approach that of nondiabetics. Conversely, CD45dimCD133+KDR+ EPCs and subpopulations coexpressing the CXCR4 receptor were not ameliorated by chronic insulin therapy, presenting the lowest levels in patients previously under insulin. Regarding oral antidiabetic drugs we were surprised to find no beneficial effect on EPCs levels, since these results differ from some published studies [43-45]. Notably, in accordance with the literature our results further demonstrated that levels of both CD45dimCD34+KDR+ and CD45dimCD133+KDR+ EPCs and even their subpopulations coexpressing the CXCR4 surface marker were inversely correlated with HbA1c, underscoring the importance of the glycemic control for EPCs response to an AMI. Taken together, these results suggest that insulin, but not oral antidiabetic drugs, may increase survival of circulating EPCs (denoted by the trend to the normalization of CD45dimCD34+KDR+ levels). So, it is tempting to speculate that the favorable clinical outcomes associated with glycemic control during AMI may be partly dependent on stimulation of EPCs-mediated neovascularization in the ischemic myocardium. However, even chronic insulin treatment seemed unable to correct the characteristic dysfunction of diabetics EPCs (here illustrated by the decrease in CD45dimCD133+KDR+ EPCs, which may represent an impairment in mobilization from bone marrow, and reduction in CXCR4+ subpopulations, denoting a possible homing dysfunction). Yet, since patients under insulin therapy had the highest HBA1c levels, it is still unknown if with a better glycemic control chronic insulin therapy could reverse EPCs dysfunction of diabetic patients and completely normalize their response to an AMI. Altogether, our results suggest that chronic hyperglycemia and not diabetes *per se*, is the responsible for impaired EPCs response of diabetic patients to myocardial ischemia.

### Limitations

The limitations of our study should be acknowledged: 1) the widespread interlaboratory variations in FACS methodology used to quantify circulating EPCs is still a problem. In this study we used a standardized protocol, which has demonstrated a high accuracy in the detection of different EPCs subpopulations with angiogenic properties and enable us to study the differentiation and commitment of these cells, from early precursors to more mature circulating EPCs [17,48]. However, we recognize that further standardization of EPCs definitions and FACS protocols would be important to better compare results between different groups; 2) the long list of exclusion criteria limited the enrollment of higher number of AMI patients in this study, resulting in a relatively small number of patients in each antidiabetic treatment group. Therefore, the data regarding the comparison of EPCs levels between the different antidiabetic treatment categories should be interpreted with caution because of the risk of error type II and further studies to explore how insulin therapy may interact and affect diabetic EPC numbers and function in patients with AMI, are obviously warranted; 3) since investigation of the molecular mechanisms regulating circulating EPCs levels in AMI diabetic patients was not under the scope of this study, the signaling pathways underlying the observed reduction in EPCs levels during the early phases of AMI in diabetic as compared to nondiabetic patients are unknown”.

## Conclusions

In summary, our data demonstrates that there is a progressive decrease in EPCs response to an AMI, according to the glycemic continuum, from NGM to pre-diabetes and finally DM, and that the exhaustion of the EPCs pool is influenced by the degree of glycemic control. Furthermore, it seems conceivable to use therapeutic interventions, such as insulin, to try to reverse the impaired response to an AMI of diabetics and possibly improve the dismal prognosis of these patients.

## Competing interests

The authors declare that they have no competing interests.

## Authors’ contributions

NA designed the study, contributed to clinical data acquisition and has written the first draft of the manuscript. RF contributed to data interpretation and critically revised the manuscript. AS helped to draft the manuscript and performed the statistical analysis. FS participated in the acquisition of clinical data. AL participated in interpretation of the data and helped to draft the manuscript. TC carried out the flow cytometry analysis and participated in interpretation of the data. AP contributed to the refinement of the research protocol, to the data analysis and interpretation, and to the development of the manuscript. GMP and LAP contributed to obtaining funding, and critically revised the manuscript. LG participated in the study design, contributed to obtaining funding and critically revised the manuscript. CFR conceived the study, participated in the data interpretation, oversaw the development of the manuscript and supervised the project. All authors read and approved the final manuscript.

## References

[B1] Harjai KJ Stone GW Boura J Mattos L Chandra H Cox D Grines L O’Neill W Grines C Comparison of outcomes of diabetic and nondiabetic patients undergoing primary angioplasty for acute myocardial infarction Am J Cardiol 2003 91 9 1041 1045 12714143 10.1016/s0002-9149(03)00145-0

[B2] Asahara T Murohara T Sullivan A Silver M van der Zee R Li T Witzenbichler B Schatteman G Isner JM Isolation of putative progenitor endothelial cells for angiogenesis Science 1997 275 5302 964 967 9020076 10.1126/science.275.5302.964

[B3] Zampetaki A Kirton JP Xu Q Vascular repair by endothelial progenitor cells Cardiovasc Res 2008 78 3 413 421 18349136 10.1093/cvr/cvn081

[B4] Hristov M Erl W Weber PC Endothelial progenitor cells: mobilization, differentiation, and homing Arterioscler Thromb Vasc Biol 2003 23 7 1185 1189 12714439 10.1161/01.ATV.0000073832.49290.B5

[B5] Urbich C Aicher A Heeschen C Dernbach E Hofmann WK Zeiher AM Dimmeler S Soluble factors released by endothelial progenitor cells promote migration of endothelial cells and cardiac resident progenitor cells J Mol Cell Cardiol 2005 39 5 733 742 16199052 10.1016/j.yjmcc.2005.07.003

[B6] Hill JM Zalos G Halcox JP Schenke WH Waclawiw MA Quyyumi AA Finkel T Circulating endothelial progenitor cells, vascular function, and cardiovascular risk N Engl J Med 2003 348 7 593 600 12584367 10.1056/NEJMoa022287

[B7] Shintani S Murohara T Ikeda H Ueno T Honma T Katoh A Sasaki K Shimada T Oike Y Imaizumi T Mobilization of endothelial progenitor cells in patients with acute myocardial infarction Circulation 2001 103 23 2776 2779 11401930 10.1161/hc2301.092122

[B8] Massa M Rosti V Ferrario M Campanelli R Ramajoli I Rosso R De Ferrari GM Ferlini M Goffredo L Bertoletti A Klersy C Pecci A Moratti R Tavazzi L Increased circulating hematopoietic and endothelial progenitor cells in the early phase of acute myocardial infarction Blood 2005 105 1 199 206 15345590 10.1182/blood-2004-05-1831

[B9] Hamed S Brenner B Aharon A Daoud D Roguin A Nitric oxide and superoxide dismutase modulate endothelial progenitor cell function in type 2 diabetes mellitus Cardiovasc Diabetol 2009 8 56 19878539 10.1186/1475-2840-8-56 PMC2773759

[B10] Fadini GP Miorin M Facco M Bonamico S Baesso I Grego F Menegolo M de Kreutzenberg SV Tiengo A Agostini C Avogaro A Circulating endothelial progenitor cells are reduced in peripheral vascular complications of type 2 diabetes mellitus J Am Coll Cardiol 2005 45 9 1449 1457 15862417 10.1016/j.jacc.2004.11.067

[B11] Tepper OM Galiano RD Capla JM Kalka C Gagne PJ Jacobowitz GR Levine JP Gurtner GC Human endothelial progenitor cells from type II diabetics exhibit impaired proliferation, adhesion, and incorporation into vascular structures Circulation 2002 106 22 2781 2786 12451003 10.1161/01.cir.0000039526.42991.93

[B12] Li H Zhang X Guan X Cui X Wang Y Chu H Cheng M Advanced glycation end products impair the migration, adhesion and secretion potentials of late endothelial progenitor cells Cardiovasc Diabetol 2012 11 46 22545734 10.1186/1475-2840-11-46 PMC3403843

[B13] DeFronzo RA Abdul-Ghani M Assessment and treatment of cardiovascular risk in prediabetes: impaired glucose tolerance and impaired fasting glucose Am J Cardiol 2011 108 3 Suppl 3B 24B 10.1016/j.amjcard.2011.03.013 21802577

[B14] Antonio N Fernandes R Rodriguez-Losada N Jimenez-Navarro MF Paiva A de Teresa GE Goncalves L Ribeiro CF Providencia LA Stimulation of endothelial progenitor cells: a new putative effect of several cardiovascular drugs Eur J Clin Pharmacol 2010 66 3 219 230 20012029 10.1007/s00228-009-0764-y

[B15] Ryden L Grant PJ Anker SD Berne C Cosentino F Danchin N Deaton C Escaned J Hammes HP Huikuri H Marre M Marx N Mellbin L Ostergren J Patrono C Seferovic P Uva MS Taskinen MR Tendera M Tuomilehto J Valensi P Zamorano JL ESC Guidelines on diabetes, pre-diabetes, and cardiovascular diseases developed in collaboration with the EASD: the Task Force on diabetes, pre-diabetes, and cardiovascular diseases of the European Society of Cardiology (ESC) and developed in collaboration with the European Association for the Study of Diabetes (EASD) Eur Heart J 2013 34 39 3035 3087 23996285 10.1093/eurheartj/eht108

[B16] American Diabetes Association Diagnosis and classification of diabetes mellitus Diabetes Care 2012 35 Suppl 1 S64 S71 22187472 10.2337/dc12-s064 PMC3632174

[B17] Schmidt-Lucke C Fichtlscherer S Aicher A Tschope C Schultheiss HP Zeiher AM Dimmeler S Quantification of circulating endothelial progenitor cells using the modified ISHAGE protocol PLoS One 2010 5 11 e13790 21072182 10.1371/journal.pone.0013790 PMC2972200

[B18] Ingram DA Caplice NM Yoder MC Unresolved questions, changing definitions, and novel paradigms for defining endothelial progenitor cells Blood 2005 106 5 1525 1531 15905185 10.1182/blood-2005-04-1509

[B19] Walter DH Haendeler J Reinhold J Rochwalsky U Seeger F Honold J Hoffmann J Urbich C Lehmann R Arenzana-Seisdesdos F Aicher A Heeschen C Fichtlscherer S Zeiher AM Dimmeler S Impaired CXCR4 signaling contributes to the reduced neovascularization capacity of endothelial progenitor cells from patients with coronary artery disease Circ Res 2005 97 11 1142 1151 16254213 10.1161/01.RES.0000193596.94936.2c

[B20] Sutherland DR Anderson L Keeney M Nayar R Chin-Yee I The ISHAGE guidelines for CD34+ cell determination by flow cytometry. International Society of Hematotherapy and Graft Engineering J Hematother 1996 5 3 213 226 8817388 10.1089/scd.1.1996.5.213

[B21] Sun JY Zhai L Li QL Ye JX Kang LN Xie J Xu B Effects of ACE inhibition on endothelial progenitor cell mobilization and prognosis after acute myocardial infarction in type 2 diabetic patients Clinics (Sao Paulo) 2013 68 5 665 673 23778412 10.6061/clinics/2013(05)14 PMC3654302

[B22] Ling L Shen Y Wang K Jiang C Fang C Ferro A Kang L Xu B Worse clinical outcomes in acute myocardial infarction patients with type 2 diabetes mellitus: relevance to impaired endothelial progenitor cells mobilization PLoS One 2012 7 11 e50739 23226370 10.1371/journal.pone.0050739 PMC3511359

[B23] Yeh ET Zhang S Wu HD Korbling M Willerson JT Estrov Z Transdifferentiation of human peripheral blood CD34+-enriched cell population into cardiomyocytes, endothelial cells, and smooth muscle cells in vivo Circulation 2003 108 17 2070 2073 14568894 10.1161/01.CIR.0000099501.52718.70

[B24] Werner N Kosiol S Schiegl T Ahlers P Walenta K Link A Bohm M Nickenig G Circulating endothelial progenitor cells and cardiovascular outcomes N Engl J Med 2005 353 10 999 1007 16148285 10.1056/NEJMoa043814

[B25] Fortunato O Spinetti G Specchia C Cangiano E Valgimigli M Madeddu P Migratory activity of circulating progenitor cells and serum SDF-1alpha predict adverse events in patients with myocardial infarction Circ Res 2013 100 2 192 200 10.1093/cvr/cvt153 23761401

[B26] Rosenson RS Reasner CA Therapeutic approaches in the prevention of cardiovascular disease in metabolic syndrome and in patients with type 2 diabetes Curr Opin Cardiol 2004 19 5 480 487 15316457 10.1097/01.hco.0000133111.66486.c6

[B27] Zhao CT Wang M Siu CW Hou YL Wang T Tse HF Yiu KH Myocardial dysfunction in patients with type 2 diabetes mellitus: role of endothelial progenitor cells and oxidative stress Cardiovasc Diabetol 2012 11 147 23217199 10.1186/1475-2840-11-147 PMC3537556

[B28] Fadini GP Sartore S Schiavon M Albiero M Baesso I Cabrelle A Agostini C Avogaro A Diabetes impairs progenitor cell mobilisation after hindlimb ischaemia-reperfusion injury in rats Diabetologia 2006 49 12 3075 3084 17072586 10.1007/s00125-006-0401-6

[B29] Marfella R Rizzo MR Siniscalchi M Paolisso P Barbieri M Sardu C Savinelli A Angelico N Del Gaudio S Esposito N Rambaldi PF D'Onofrio N Mansi L Mauro C Paolisso G Balestrieri ML Peri-procedural tight glycemic control during early percutaneous coronary intervention up-regulates endothelial progenitor cell level and differentiation during acute ST-elevation myocardial infarction: effects on myocardial salvage Int J Cardiol 2013 168 4 3954 3962 23876463 10.1016/j.ijcard.2013.06.053

[B30] Chen MC Chen CJ Yang CH Liu WH Fang CY Hsieh YK Chang HW Relationship of the percentage of circulating endothelial progenitor cell to the severity of coronary artery disease Heart Vessels 2008 23 1 47 52 18273546 10.1007/s00380-007-1006-9

[B31] Bozdag-Turan I Turan RG Turan CH Ludovicy S Akin I Kische S Arsoy NS Schneider H Ortak J Rehders T Hermann T Paranskaya L Kohlschein P Bastian M Ulus AT Sahin K Ince H Nienaber CA Relation between the frequency of CD34(+) bone marrow derived circulating progenitor cells and the number of diseased coronary arteries in patients with myocardial ischemia and diabetes Cardiovasc Diabetol 2011 10 107 22118372 10.1186/1475-2840-10-107 PMC3235974

[B32] Vasa M Fichtlscherer S Aicher A Adler K Urbich C Martin H Zeiher AM Dimmeler S Number and migratory activity of circulating endothelial progenitor cells inversely correlate with risk factors for coronary artery disease Circ Res 2001 89 1 E1 E7 11440984 10.1161/hh1301.093953

[B33] Khan SS Solomon MA McCoy JP Jr Detection of circulating endothelial cells and endothelial progenitor cells by flow cytometry Cytometry B Clin Cytom 2005 64 1 1 8 15668988 10.1002/cyto.b.20040

[B34] Yoder MC Human endothelial progenitor cells Cold Spring Harb Perspect Med 2012 2 7 a006692 22762017 10.1101/cshperspect.a006692 PMC3385946

[B35] Handgretinger R Gordon PR Leimig T Chen X Buhring HJ Niethammer D Kuci S Biology and plasticity of CD133+ hematopoietic stem cells Ann N Y Acad Sci 2003 996 141 151 12799292 10.1111/j.1749-6632.2003.tb03242.x

[B36] Peichev M Naiyer AJ Pereira D Zhu Z Lane WJ Williams M Oz MC Hicklin DJ Witte L Moore MA Rafii S Expression of VEGFR-2 and AC133 by circulating human CD34(+) cells identifies a population of functional endothelial precursors Blood 2000 95 3 952 958 10648408

[B37] Yamaguchi J Kusano KF Masuo O Kawamoto A Silver M Murasawa S Bosch-Marce M Masuda H Losordo DW Isner JM Asahara T Stromal cell-derived factor-1 effects on ex vivo expanded endothelial progenitor cell recruitment for ischemic neovascularization Circulation 2003 107 9 1322 1328 12628955 10.1161/01.cir.0000055313.77510.22

[B38] Barr EL Zimmet PZ Welborn TA Jolley D Magliano DJ Dunstan DW Cameron AJ Dwyer T Taylor HR Tonkin AM Wong TY McNeil J Shaw JE Risk of cardiovascular and all-cause mortality in individuals with diabetes mellitus, impaired fasting glucose, and impaired glucose tolerance: the Australian Diabetes, Obesity, and Lifestyle Study (AusDiab) Circulation 2007 116 2 151 157 17576864 10.1161/CIRCULATIONAHA.106.685628

[B39] Fadini GP Pucci L Vanacore R Baesso I Penno G Balbarini A Di Stefano R Miccoli R de Kreutzenberg S Coracina A Tiengo A Agostini C Del Prato S Avogaro A Glucose tolerance is negatively associated with circulating progenitor cell levels Diabetologia 2007 50 10 2156 2163 17579827 10.1007/s00125-007-0732-y

[B40] Dong L Kang L Ding L Chen Q Bai J Gu R Li L Xu B Insulin modulates ischemia-induced endothelial progenitor cell mobilization and neovascularization in diabetic mice Microvasc Res 2011 82 3 227 236 21964072 10.1016/j.mvr.2011.09.006

[B41] Zhao L Cao F Yin T Sun D Cheng K Zhang J Wang H Moderate dose insulin promotes function of endothelial progenitor cells Cell Biol Int 2011 35 3 215 220 21143206 10.1042/CBI20100205

[B42] Humpert PM Djuric Z Zeuge U Oikonomou D Seregin Y Laine K Eckstein V Nawroth PP Bierhaus A Insulin stimulates the clonogenic potential of angiogenic endothelial progenitor cells by IGF-1 receptor-dependent signaling Mol Med 2008 14 5–6 301 308 18309377 10.2119/2007-00052.Humpert PMC2255559

[B43] Wang CH Ting MK Verma S Kuo LT Yang NI Hsieh IC Wang SY Hung A Cherng WJ Pioglitazone increases the numbers and improves the functional capacity of endothelial progenitor cells in patients with diabetes mellitus Am Heart J 2006 152 6 1051 e1051-1058 10.1016/j.ahj.2006.07.029 17161050

[B44] Fadini GP Boscaro E Albiero M Menegazzo L Frison V de Kreutzenberg S Agostini C Tiengo A Avogaro A The oral dipeptidyl peptidase-4 inhibitor sitagliptin increases circulating endothelial progenitor cells in patients with type 2 diabetes: possible role of stromal-derived factor-1alpha Diabetes Care 2010 33 7 1607 1609 20357375 10.2337/dc10-0187 PMC2890368

[B45] Pistrosch F Herbrig K Oelschlaegel U Richter S Passauer J Fischer S Gross P PPARgamma-agonist rosiglitazone increases number and migratory activity of cultured endothelial progenitor cells Atherosclerosis 2005 183 1 163 167 15907852 10.1016/j.atherosclerosis.2005.03.039

[B46] Yue WS Lau KK Siu CW Wang M Yan GH Yiu KH Tse HF Impact of glycemic control on circulating endothelial progenitor cells and arterial stiffness in patients with type 2 diabetes mellitus Cardiovasc Diabetol 2011 10 113 22185563 10.1186/1475-2840-10-113 PMC3258289

[B47] Churdchomjan W Kheolamai P Manochantr S Tapanadechopone P Tantrawatpan C U-pratya Y Issaragrisil S Comparison of endothelial progenitor cell function in type 2 diabetes with good and poor glycemic control BMC Endocr Disord 2010 10 5 20374643 10.1186/1472-6823-10-5 PMC2858721

[B48] Estes ML Mund JA Mead LE Prater DN Cai S Wang H Pollok KE Murphy MP An CS Srour EF Ingram DA Jr Case J Application of polychromatic flow cytometry to identify novel subsets of circulating cells with angiogenic potential Cytometry A 2010 77 9 831 839 20803735 10.1002/cyto.a.20921 PMC2931367

